# USP25 in atherosclerosis: a new kid on the block

**DOI:** 10.1016/j.ebiom.2026.106243

**Published:** 2026-03-31

**Authors:** Iqra Ilyas, Suowen Xu

**Affiliations:** a Department of Endocrinology and Metabolism, The First Affiliated Hospital of USTC, Division of Life Sciences and Medicine, University of Science and Technology of China, Hefei, 230027, China; b Department of Pharmacology, Max Planck Institute for Heart and Lung Research, Ludwigstr. 43, Bad Nauheim, 61231, Germany; c Anhui Provincial Key Laboratory of Metabolic Health and Panvascular Diseases, Hefei, China

Atherosclerosis is the primary pathological driver of cardiovascular diseases, including myocardial infarction and stroke, which are leading causes of global morbidity and mortality.^1^ Although lipid accumulation and chronic vascular inflammation are recognised hallmarks of atherosclerosis, the molecular regulators that integrate inflammatory signalling with vascular cell dysfunction remain unclear. In a recent study published in *eBioMedicine*, Su et al. identified the deubiquitinating enzyme USP25 (ubiquitin-specific peptidase 25) as an important negative regulator of vascular inflammation and atherosclerotic progression.^2^ Their work provides mechanistic insight into how ubiquitin-dependent signalling pathways shape inflammatory responses in atherosclerosis and highlights USP25 as a potential therapeutic target.

The authors demonstrated that USP25 expression was significantly reduced in human atherosclerotic plaques compared with normal vascular tissues. Using both global and haematopoietic cell-specific USP25-deficient mice, they further showed that USP25 deficiency exacerbates atherosclerotic lesion development, accompanied by increased lipid deposition, macrophage accumulation, and lesional inflammation. Notably, USP25 deficiency did not alter the serum lipid profile in these mice,^2^ suggesting that its protective effect is independent of lipid lowering. Mechanistically, the study identified receptor-interacting protein kinase 1 (RIPK1) as the key substrate and downstream target of USP25. RIPK1 is a signalling molecule known for its roles in cell survival, apoptosis, and necroptosis. RIPK1 functions upstream of NF-κB and MAPK signalling pathways and has been identified as a pro-atherosclerotic factor.^3^ USP25 regulates RIPK1 activation by cleaving K63-linked ubiquitin chains on RIPK1. This reduces the production of pro-inflammatory cytokines and inhibits vascular inflammation. Importantly, RIPK1 downregulation via AAV-Ripk1-shRNA in ApoE^−/−^ bone marrow chimeric mice mitigated the exacerbated atherosclerosis induced by USP25 deficiency. This suggests that USP25 ameliorates atherosclerosis by modulating RIPK1. Conversely, USP25 overexpression in haematopoietic cells reduced lesion area, necrotic core formation, lipid accumulation, and macrophage infiltration in the aortic root of ApoE^−/−^ mice fed a high-fat diet.^2^

Recently, the role of ubiquitin-specific proteases (USPs) in atherosclerosis has attracted increasing research interest. Several USP family members have been implicated in atherosclerosis, including USP53,^4^ USP9X,^5^ USP22,^6^ USP5,^7^ and USP11.^8^ Among these, USP53, USP9X and USP22 are considered atheroprotective,^4^, ^5^, ^6^ whereas USP5 and USP11 have been associated with pro-atherogenic effects.^7^^,^^8^ However, the biological functions of USPs appear to be highly context-dependent and vary according to cell type and signalling environment. Therefore, a systematic investigation of ubiquitin-modifying enzymes in vascular biology could reveal additional regulators of plaque development and instability. For a broader discussion of USP family members in atherosclerosis, readers may refer to a recent comprehensive review.^9^

The study by Su et al. expands our understanding of how post-translational modifications govern inflammatory pathways within atherosclerotic lesions by identifying USP25 as a new player in atherosclerosis.^2^ Furthermore, this study provides a molecular framework linking dysregulated ubiquitination to the pathophysiology of vascular inflammation.^2^ From a clinical perspective, this study meaningfully contributes to the evolving paradigm of atherosclerosis management.^2^ Contemporary therapies primarily focus on reducing lipid levels and systemic inflammation. Statins, ezetimibe, PCSK9 inhibitors, ACLY inhibitors, and anti-inflammatory agents, such as colchicine, have demonstrated clinical benefits, highlighting the importance of targeting inflammatory pathways in cardiovascular disease. However, many patients continue to experience residual cardiovascular risk despite receiving optimal lipid-lowering therapy. Identification of USP25 as a regulator of RIPK1-driven inflammation suggests that targeting ubiquitination-dependent signalling pathways could complement current therapies to mitigate residual inflammation.

The mechanistic insights provided by the authors also have broader implications for future basic research. The finding that USP25 restrains RIPK1 activity in the context of atherosclerosis suggests that vascular inflammation may be tightly regulated by deubiquitinating enzymes. This raises the possibility that other members of the USP family could similarly influence plaque development and stability. Interestingly, Su et al. identified reduced expression of four USPs in human carotid artery plaque datasets including, USP47, USP25, USP16 and USP9X. While USP47 protein levels remained unchanged in human and mouse atherosclerotic lesions, USP9X has already been reported to protect against atherosclerosis by reducing foam cell formation and inflammation in macrophages.^5^ It is therefore plausible that USP16, similar to USP25 and USP9X, may also exert vascular resilient and atheroprotective effects, although this remains to be experimentally validated.

Another important strength of this study is the integration of human tissue analysis with multiple *in vivo* approaches. The observation that USP25 expression is reduced in human atherosclerotic plaques enhances the translational relevance of the experimental findings. Such approaches are essential for bridging the gap between molecular discoveries and potential therapeutic applications. Future studies should determine whether altered USP25 expression correlates with plaque instability, clinical outcomes, or response to existing therapies.

Despite these strengths, several important questions remain unresolved. First, the mechanisms responsible for USP25 downregulation in human atherosclerotic lesions were not fully explored. Defining the upstream regulatory pathways controlling USP25 expression may uncover additional therapeutic opportunities. Second, both global and haematopoietic USP25 deficiency increased necrotic core formation. Because necrotic core formation is closely linked to impaired efferocytosis, it remains unclear whether haematopoietic USP25 deficiency influences efferocytosis and thereby contributes to plaque progression. Third, the cell-type-specific functions of USP25 within atherosclerotic lesions require further clarification. Atherosclerosis involves multiple vascular and immune cell populations, including endothelial cells, macrophages, and smooth muscle cells. Determining how USP25 regulates RIPK1 signalling across these distinct cell types may help explain the complex inflammatory dynamics within plaques. Because most currently characterised USP family members in atherosclerosis are studied predominantly in macrophages, whether additional USPs in endothelial cells or smooth muscle cells also regulate plaque biology remains largely unknown and represents an important future direction for the field. Fourth, although inhibition of RIPK1-mediated inflammation appears promising, the safety and feasibility of targeting this pathway in cardiovascular disease remain uncertain. RIPK1 plays essential roles in host defence and cell survival,^10^ and systemic inhibition may therefore produce unintended adverse effects. Accordingly, future therapeutic strategies may need to focus on selectively modulating USP25 or related ubiquitination pathways in vascular tissues rather than broad systemic RIPK1 inhibition.

In summary, the study by Su et al. provides important insights into the molecular regulation of vascular inflammation by identifying USP25 as a negative regulator of RIPK1-mediated signalling in atherosclerosis. By linking ubiquitin-dependent regulation to inflammatory pathways, this study advances our understanding of the molecular mechanisms driving plaque development. These findings highlight the therapeutic potential of targeting ubiquitination networks to modulate vascular inflammation and reduce cardiovascular risk, offering valuable direction for both basic researchers and clinicians. Continued exploration of deubiquitinating enzymes in vascular biology may ultimately lead to novel anti-atherosclerotic therapies beyond current lipid-lowering strategies.

## Contributors

SX conceptualised this paper. II wrote the original draft. Both authors read and approved the final manuscript.

## Declaration of interests

All the authors declare that they have no competing interests.
